# Tip-Enhanced Raman Spectroscopy Based on Spiral Plasmonic Lens Excitation

**DOI:** 10.3390/s22155636

**Published:** 2022-07-28

**Authors:** Kai Gu, Ming Sun, Yang Zhang

**Affiliations:** School of Electronic and Optical Engineering, Nanjing University of Science and Technology, Nanjing 210094, China; gk0730@njust.edu.cn (K.G.); 218104010144@njust.edu.cn (Y.Z.)

**Keywords:** plasmonic lens, surface plasmon polariton, tip-enhanced Raman spectroscopy, finite-difference time domain

## Abstract

In this study, we proposed the idea of replacing the traditional objective lens in bottom-illumination mode with a plasmonic lens (PL) to achieve tip-enhanced Raman spectroscopy (TERS). The electric field energy of surface plasmon polaritons (SPPs) of the spiral PL was found to be more concentrated at the focal point without any sidelobe using the finite-difference time domain (FDTD) method compared with that of a symmetry-breaking PL. This property reduces far-field background noise and increases the excitation efficiency of the near-field Raman signal. The disadvantage of only the near-field Raman scattering of samples at the center of the structure being detected when using an ordinary PL in TERS is overcome by using our proposed method of changing only the polarization of the incident light.

## 1. Introduction

Although scanning near-field optical microscopy (SNOM) and atomic force microscopy (AFM) are used to break the diffraction limit for imaging various sample surfaces at high resolution, their chemical and biological information at the nanometer scale cannot be determined. To analyze vibrational spectroscopy, the chemical or biological properties of samples are studied using methods such as Raman spectroscopy, a nondestructive and powerful tool that is widely used for the detection of molecular compositions, chemical bonds, and crystal structures [1,2,3]. Compared to crystalline materials, the majority of samples, such as biomolecules, are weak Raman scatterers, that is, their Raman scattering cross section is very small [4,5,6]. To significantly enhance the intensity and resolution of the Raman scattering signal, near-field optical microscopy is combined with Raman scattering technology to form a TERS system [7,8,9,10]. TERS arises from the strong enhancement of the electromagnetic field occurring at the apex of a sharp noble-metal tip when irradiated by an incident light with strong longitudinal components due to the excitation of localized surface plasmons (LSPs) and the lightning rod effect [11,12]. The sharp metal tip, placed in close proximity to the sample, acts as an active probe, efficiently enhancing the localized field and scattering Raman emission [13,14,15]. The metalized AFM probe therefore acts as the most commonly used metal nanoantenna.

Three different modes of excitation are used in TERS: bottom, side, and top illumination [16,17,18]. Side and top illumination are ideal for large, opaque samples with considerable thicknesses, whereas bottom illumination is perfect for transparent samples. Bottom illumination is preferred when all three types meet the requirements because it usually uses an oil lens as the focusing lens. This can not only increase the strength of the longitudinal electric field (the direction of the electric field is parallel to the axis of the tip) that contributes to the enhanced local electric field formed when the tip is above the focal point but makes the focal point as small as possible. Therefore, the large far-field imaging noise due to a large spot size (produced using an objective lens with a relatively small numerical aperture and a long working distance in side-illumination mode) is reduced in bottom-illumination mode. The problem associated with top-illumination TERS that the contrast is close to the noise level because the generated Raman signal is blocked by the cantilever does not occur in bottom-illumination mode, so bottom-illumination mode has been widely used in a variety of situations. To further improve the Raman intensity, the incident light is radially polarized, which produces a single solid focal point in the *z*-direction electric field. In contrast, a linearly polarized light forms a focus in the *z*-direction electric field of two lobes. The *z*-electric field intensity at the focal point is only half that of the radially polarized light, reducing the enhancement effect and sensitivity in TERS [19]. To further improve the Raman intensity, a perfect radially polarized beam is used [20,21]. However, a complex optical system is required, irrespective of the type of light used. The desired enhancement of Raman scattering is achieved only when the excitation light and the objective lens are strictly aligned [4]. In bottom-illumination TERS, instead of a conventional optical lens, a novel metallic plasmonic lens (PL) is used. Mingqian Zhang et al. [22] generated the Raman spectrum of single-walled carbon nanotubes using a PL with symmetry-breaking, semi-annular slits corrugated on a gold film to TERS under linearly polarized beam illumination. However, they only detected the sample at the focal point at the center of the structure and therefore obtained the near-field Raman scattering signal only at this point.

In this study, we designed a spiral PL to generate a single surface plasmon polariton (SPP) focal point and plasmonic vortex using left- (LCP) and right-handed circular polarization (RCP) illumination, respectively. This method not only reduces the far-field background noise by eliminating sidelobes at the focal point and enhances the excitation efficiency of the near-field Raman signal [22] but also achieves a near-field Raman scattering signal at other points of the sample (at the plasmonic vortex) by changing the position of the tip and the polarization of the incident light.

## 2. Methods

**Figure 1:**
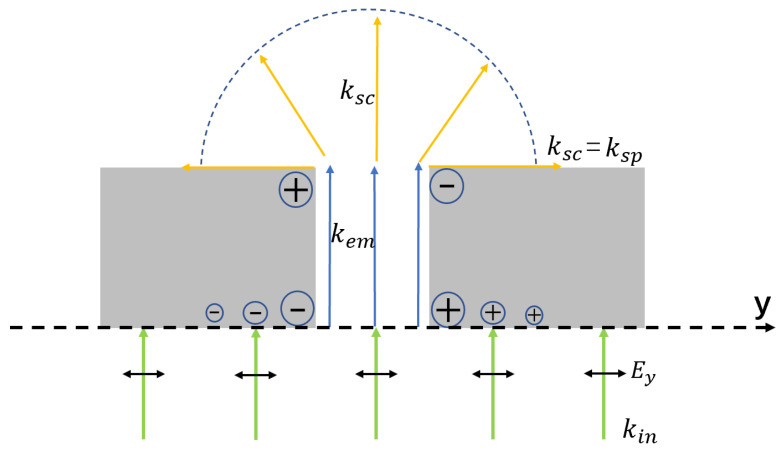
Schematic diagram of SPPs excited by the metal slit.

A PL works on the principle of excitation of SPPs by metal slits, as shown in Figure 1. When the wavelength of the incident light is more than twice the width of the slit, no transverse electric (TE) waves (*E* is parallel to the slit long axis) propagate through the slit. However, according to the electromagnetic wave equation in matter, *Jy* = σ*Ey*, where *Jy* is the current density, σ is the electrical conductivity, and *Ey* is the electric field intensity, a very low electric field intensity yields a very high current density due to the high conductivity of the metallic material when TM waves (*E* is vertical to the slit long axis) incident on a metal, which is considered an ideal conductor with no electromagnetic field. According to the boundary conditions, *Jy* is equal to the surface magnetic field intensity (*Hy*), and the surface charge density (ρ) is equal to the inductance perpendicular to the surface (*Dy*). *Jy* stops abruptly at the edges of the slit, causing an accumulation of charges at the sharp corners, and these charges on opposite edges of the slit behave like an electric dipole [23,24,25,26]. Because these dipoles oscillate with the incident wave, they act as light sources emitting photons ($k_{em}$). Inside the slit, the surface charges and currents carry these photons in the *z* direction, disrupting the movement of charges at the exit edges and giving rise to another large dipole. These charges are accumulated at the upper and lower corners of the slit during the time-harmonic steady state. The electromagnetic waves radiated by the dipoles oscillating on the lower surface are diffracted when they propagate to the slit on the upper surface, and electromagnetic waves with a wave vector equal to that of the SPP are generated ($k_{sc}$), which ultimately excite the SPP propagating along the metal surface; therefore, the edge of the slit is considered a line of SPP point sources. The normal incidence of light generates an equal phase distribution of electron oscillations across the slit, and the propagation direction of SPPs generated by the slit is always perpendicular to the slit. In other words, the direction for SPPs coincides with the polarization direction of the illuminating beam because SPPs are generated only by the polarization component that is perpendicular to the slit. Furthermore, the projected component of the electric field in the propagation direction of SPPs excites SPPs if the polarization direction of the incident light is not perpendicular to the slit.

**Figure 2:**
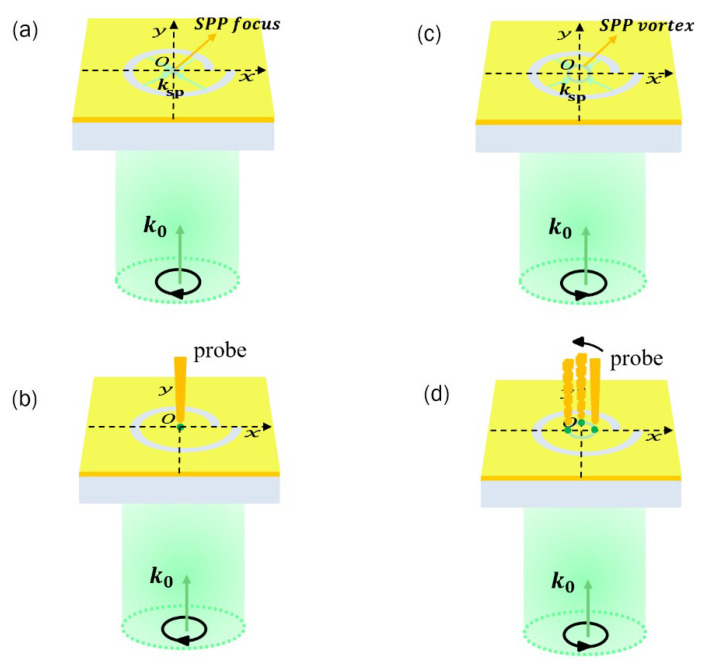
Formation of the (**a**) SPP focus and (**c**) SPP vortex when the incident light is LCP and RCP, respectively. Formation of the enhanced electric field (**b**) when the probe is above the SPP focus and (**d**) at different positions when the probe is located at different positions above the SPP vortex.

In curved slits, each SPP point source on the curve is excited only by the normal incident light, with polarization component perpendicular to the slit. The PL is obtained using different structures, such that the SPP can achieve constructive interference somewhere in the structure to form the focus of the SPP. In other words, an SPP with a shorter wavelength and a strong longitudinal electric field that dominates the excitation field can be focused by a PL into a smaller subwavelength spot size. The geometry of a PL with spiral slits is *r* = *a* + (*λ*_SPP_*m* ∅)/2*π* [27], where *r* is the distance between the center of the PL to the slit with the azimuthal angle ∅; *a* is the inner radius of the PL; *λ*_SPP_ is the effective wavelength of the SPP; and *m* is the geometric charge of the PL, which is taken as 1. The spin angular momentum of the circularly polarized beam is *σ* = *s*ℏ, where *s* = +1 for RCP, and *s* = −1 for LCP. The polarization state of the circularly polarized beam changes over time, but its amplitude is constant. At a certain moment, the SPP point source on the spiral slit (the connecting line between the point and the center of the structure is along the polarization direction of the incident light) emits SPP waves propagating along the polarization direction of the incident light. In the next moment, the polarization state of the incident light is changed to another direction, whereas the amplitude remains unchanged, so there is another point source on the spiral slit to complete the above process. Other points also emit SPPs excited by the projection of an electric field that is perpendicular to the slit and that is also the propagation direction of SPPs. When LCP is the source of excitation, the SPP waves with the same amplitude emitted by each point are at the same distance from the center after one rotation of the polarization state of the circularly polarized beam. After some time, the SPP waves emitted by each point source can interfere at the center and form a focal point with strong longitudinal electric field components, as shown in Figure 2a. The final effect is equivalent to that a radially polarized beam excites SPPs by illuminating each point of the circular groove in the metal film.

Figure 2c shows a case in which the incident beam is RCP, where the SPP waves with the same amplitude traversing toward the center emitted by point sources at both ends of the diameter are no longer the same distance from the center after one rotation of the polarization state of circularly polarized beams, but will be different by *λ*_SPP_. Thus, an SPP vortex with a radius of approximately *λ*_SPP_/2 is formed at the center of the structure. The total topological charge of the SP vortex is described by *q* = *m* + *s*, where *q* = 1 − 1 = 0 for LCP, that is, the formation is a zero-order Bezier beam with a focused spot; and *q* = 1 + 1 = 2 for RCP, that is, the formation is a second-order Bessel beam, the size of which is the solution of second-order Bessel function of the first kind [28].

When the metal tip is located very close above the SPP focus/vortex, the external electric field polarized along the tip axis drives the free electrons in the tip to periodically move up and down along the tip axis at the same frequency as the excitation field. Because the surface area near the apex of the tip is very small, electrons generate considerable surface charge accumulation at the tip of the probe. The generation of these charges can generate a considerably increased local electric field under the tip. In addition, when the tip is close to the sample stage, the “gap plasmon” coupling effect can further enhance the local electric field and ultimately provide a strong excitation source for TERS [29].

Let us consider a circularly polarized light with a given polarization incident on the spiral PL, forming the SPP focus. This generates a strong local electric field when a probe is placed above the focus, which enhances the near-field Raman scattering signal under the probe, as shown in Figure 2b. When its polarization changes, the SPP focus becomes the SPP vortex, and the longitudinal component is still dominant in the electric field on the vortex. Therefore, when the probe is placed on a point above the vortex, a strong local electric field is formed, which enhances the near-field Raman scattering signal under the probe. By moving the probe to other points on the vortex, the near-field Raman scattering signals at different points on the sample can be detected, as shown in Figure 2d.

In this study, we used the finite-difference time domain (FDTD) method to calculate the intensity of the electric field under different conditions. The results are analyzed to evaluate the enhancement and distribution of near-field Raman scattering signals in TERS. For all simulations, we used a Yee cell with a side length of 3 nm and perfectly matched layer boundary conditions on all boundaries. The monitor was placed 5 nm above the PL surface to detect the near-field electric field intensity.

## 3. Results and Discussion

**Figure 3:**
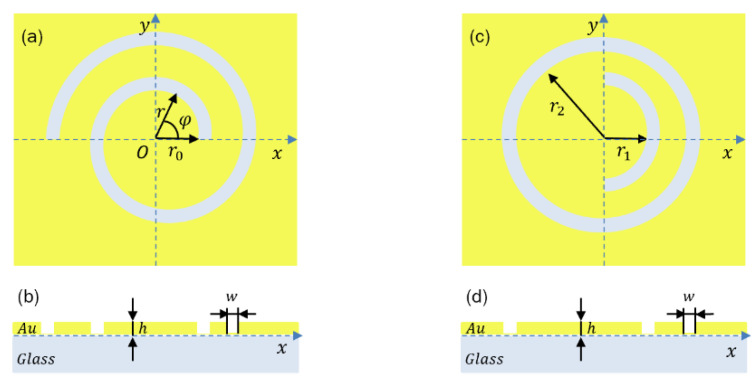
Schematic diagram of the PL structure consisting of a circle of one-and-a-half turns of (**a**,**b**) spiral slits and (**c**,**d**) symmetry-breaking circular slits.

Figure 3a,b shows a schematic of the spiral PL on a gold film supported by a glass substrate. Its initial radius (*r*_0_) is 600 nm, and its thickness (*h*) and width (*w*) are both 200 nm. The thickness of the gold film is 200 nm, which is enough to block direct transmission of the incident light to reduce the perturbation of the far-field Raman scattering signal.

**Figure 4:**
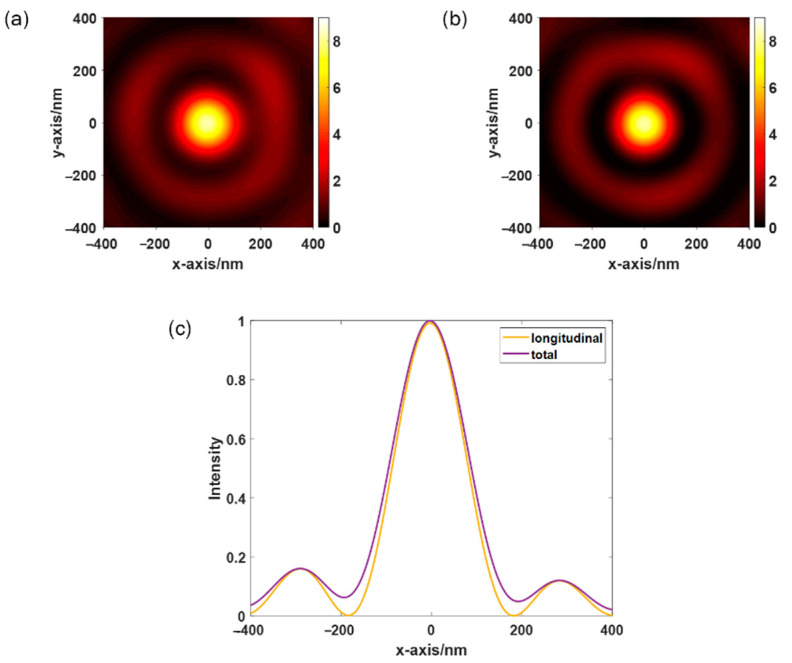
Distribution of (**a**) total electric field strength, (**b**) longitudinal electric field strength, and (**c**) normalized total electric field strength and longitudinal electric field strength along y = 0.

Figure 4a,b shows the total near-field intensity distribution and the longitudinal electric field intensity distribution, respectively, obtained by FDTD under the incident light of LCP with a wavelength of 532 nm. Figure 4c shows the normalized total electric field strength and longitudinal electric field strength along y = 0. Figure 4c shows that the main component of the focused electric field is polarized in the longitudinal direction, which confirms the applicability of the spiral PL to TERS.

**Figure 5:**
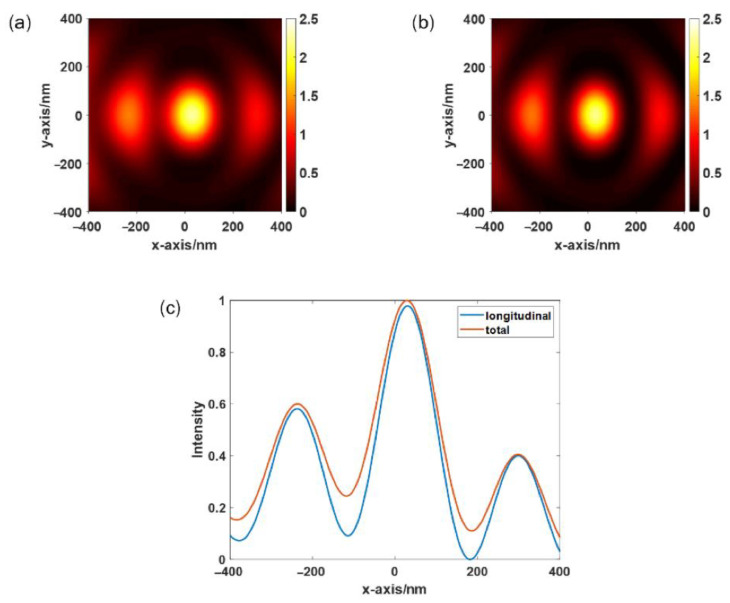
Distribution of (**a**) total electric field strength, (**b**) longitudinal electric field strength, and (**c**) normalized total electric field strength and longitudinal electric field strength along y = 0.

Figure 3c,d shows the schematic geometry of the symmetry-breaking PL with a concentric semi-annular and one annular nano-slit(s) corrugated on a gold film supported by a glass substrate. As the wavelength of the linearly polarized beam is 532 nm and the additional outer semi-annular slit with spacing, r_2_–r_1_, is 233 nm, approximately half the wavelength of the SPP wave propagates on the air–gold interface. To compare the results with the excitation effect of a spiral PL under the same conditions, the film thickness (*h*) and slit width (*w*) are set to be consistent with the spiral PL. Figure 5a,b shows the total and longitudinal near-field intensity distributions simulated in the *x*-direction linearly polarized beams as incident light by FDTD. Figure 5c shows the normalized total electric field strength and longitudinal electric field strength along y = 0. The results show that the main component of the electric field is polarized in the longitudinal direction at the center focusing position. However, when using this symmetry-breaking structure for SPP focusing, the electrical field intensity distributions are composed of a slightly off-center central focusing region and a high-energy sidelobe, which may result in large background noise. In contrast, Figure 4 shows that the electric field intensity at the SPP focus formed by spiral focusing is stronger because there is no sidelobe effect. Furthermore, smaller far-field background noise is obtained when a spiral PL is used for TERS because the focus area is reduced.

**Figure 6:**
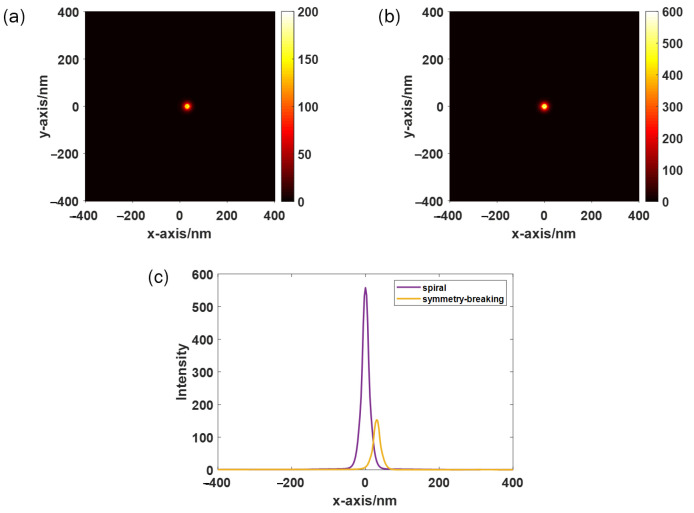
Distribution of the local electric field intensity when the probes are located 10 nm above the focus formed by the (**a**) symmetry-breaking PL and (**b**) spiral PL. (**c**) Distribution of normalized total electric field strength and longitudinal electric field strength along y = 0.

When the Raman shifts are small, the enhanced Raman scattering intensity from below the metallic probe is approximately proportional to the fourth power of the electric field enhancement [30,31]. Therefore, the large enhancement factor in TERS is associated with the large local field enhancement. The strength of the local electric field between the tip and the substrate and the corresponding TERS enhancement are dependent on many factors, such as the size and shape of the tip, the materials of the tip and the substrate, the distance between the tip and the substrate, etc., [32,33]. A sharp metal tip or a small metal particle on top of the tip can be used as an enhancement source; hence, the metal tip is simplified as an approximate model, for example, a gold particle with a radius of 50 nm. Previous studies have proven that these factors have an impact on the enhancement of the local electric field, so these parameters are set as constant. Figure 6a,b shows the electric field strength distributions when the monitors are located 5 nm above the surface of a symmetry-breaking PL and spiral PL, respectively, when the gold particle is 10 nm from the PL surface. Figure 6c shows the intensity curve along y = 0 intuitively, indicating that the near-field local electric field intensity is three times stronger with the spiral PL than with the PL with symmetry-breaking semi-annular slits. Therefore, the excitation efficiency of near-field Raman signal is improved by almost one order in TERS under the same incident light intensity.

**Figure 7:**
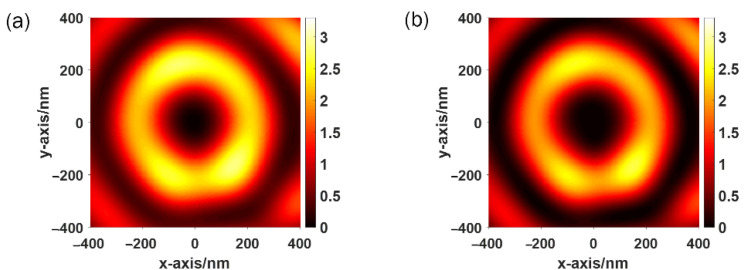
Distribution of (**a**) total electric field strength, and (**b**) longitudinal electric field strength when the incident light is RCP.

The distribution of total electric field intensity and longitudinal electric field intensity when the incident beam is converted to RCP are shown in Figure 7a,b. The SPP focus changes to a plasmonic vortex with a total topological charge of two, the radius of which is approximately *λ*_SPP_/2, which is equal to 230 nm, as shown in Figure 7. The results show that the total and longitudinal electric field strength on the plasmonic vortex are relatively uniform, and the main component of the total electric field is polarized in the longitudinal direction.

**Figure 8:**
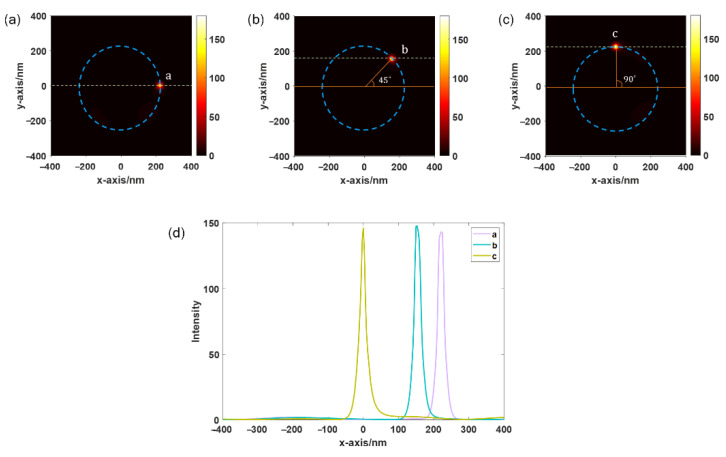
Distribution of local electric field strength when the probes are located above the SPP vortex and the angle between the line between the point on the vortex and the center of the vortex and the x-axis are (**a**) 0°, (**b**) 45°, and (**c**) 90°. The circular dotted line indicates the shape of the SPP vortex. (**d**) Distribution of the local electric field along the dashed line in the three cases.

As shown in Figure 8, when the gold particle is located above the SPP vortex produced by the spiral PL and the angle between the line between the point on the vortex and the center of the vortex and the x-axis are 0°, 45°, and 90°, the optical field is confined to a small area below the gold particle, forming gap-mode plasmons. The circular dotted line in Figure 8a–c indicates the shape of the SPP vortex in Figure 7. Therefore, the sample is sandwiched between the gold particles and the substrate, leading to an additional strong enhancement of the Raman scattering optical signal. Figure 8d shows the local electric field distribution along the dotted lines in Figure 8a–c. Nearly the same strong near-field local electric field enhancement is obtained, which ensures the stability and accuracy of the near-field-enhanced Raman scattering signal at specific points of the sample under the condition of moving gold particles.

## 4. Conclusions

In this study, we proposed an excitation method for generating longitudinal electric field components in TERS using a PL focusing SPPs, which reduced the complexity and alignment requirements of the excitation system. Using the FDTD method, we verified that the proposed spiral PL resolves the sidelobe issue at the focal point generated by the symmetry-breaking PL, thereby reducing the background noise and improving the excitation efficiency of TERS. When the incident light is converted from LCP to RCP, the SPP focus is changed to a plasmonic vortex. Therefore, in TERS, by changing the position of the probe, the near-field Raman scattering signals at different positions on the sample can be obtained, solving the shortcoming that only the near-field Raman scattering of samples at the center of the structure can be detected when using an ordinary PL. As the PL is etched on metals, it can be used as a substrate with a scanning tunneling microscope (STM) to form STM-TERS devices that operate under low-temperature and ultra-high vacuum conditions with higher system stability and higher resolution than AFM-TERS.

## Data Availability

Not applicable.
